# Supplementary material for: Extremely Low-Frequency Electromagnetic Fields Increase Cytokines in Human Hair Follicles through Wnt/β-Catenin Signaling
Source: Biomedicines. 2022 Apr 18;10(4):924. doi: 10.3390/biomedicines10040924 (PMC9024517; doi:10.3390/biomedicines10040924)
Supplement: Supplementary file 1 [file biomedicines-10-00924-s001.zip › biomedicines-1597110-supplementary.pdf]

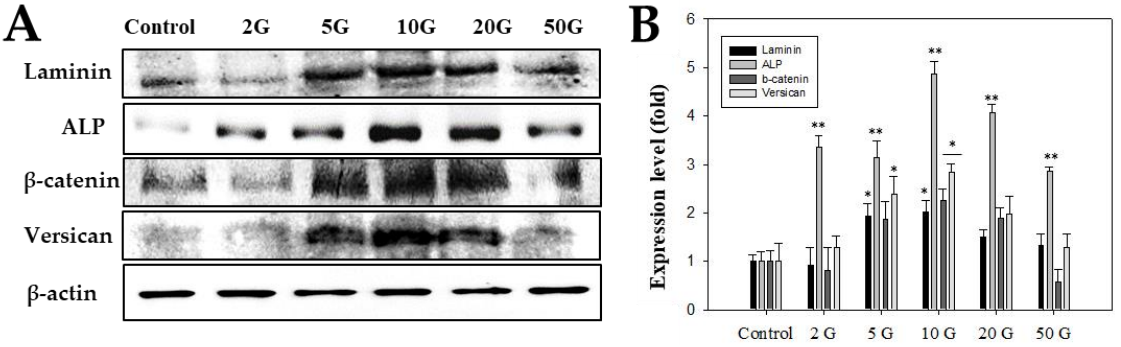

Figure S1. (A) Protein expression levels detected in human hair follicles (HFs) treated with ELF-EMF. Protein expression of β-catenin, Wnt3α, ALP, and versican, using β-actin as an internal control. (B)Relative expression intensities were calculated using ImageJ. Each bar represents the mean ± standard error of independent experiments performed in triplicate ($n = 3$). Significant differences were determined by one-way ANOVA with Tukey's post hoc test ($*P < 0.05$, $**P < 0.01$).
